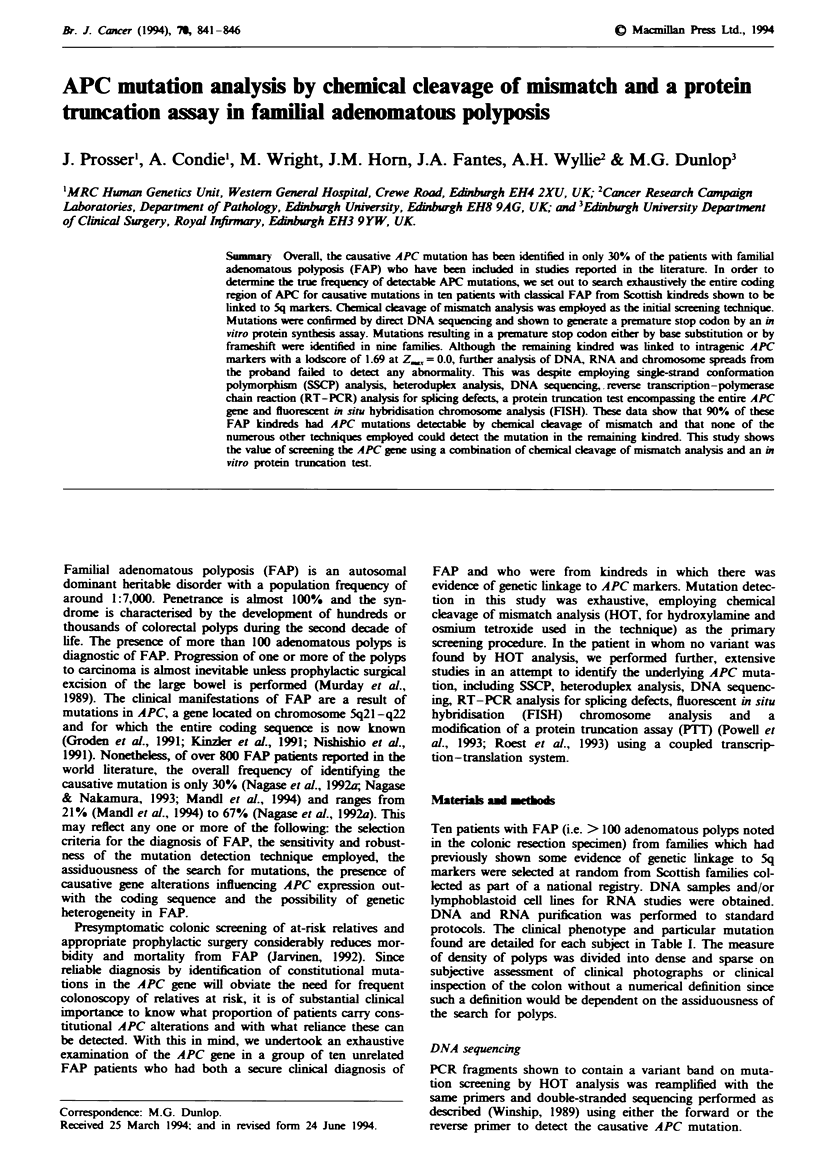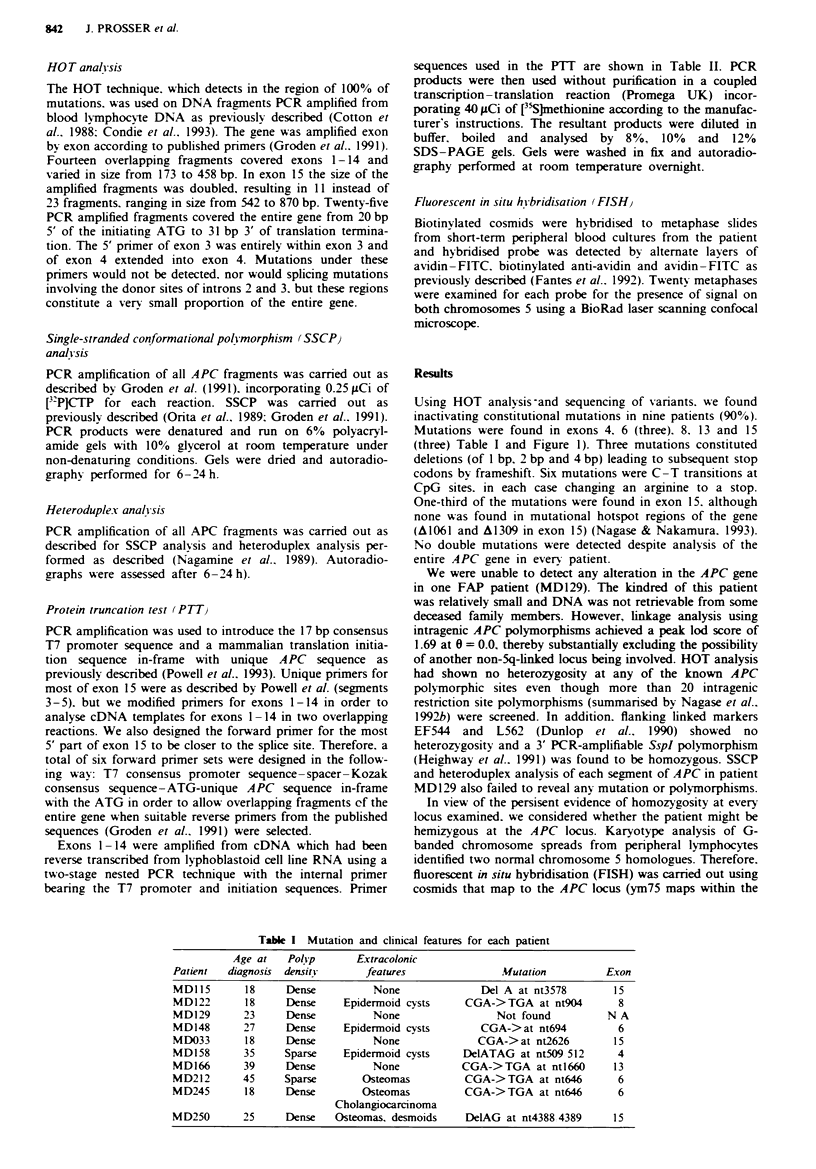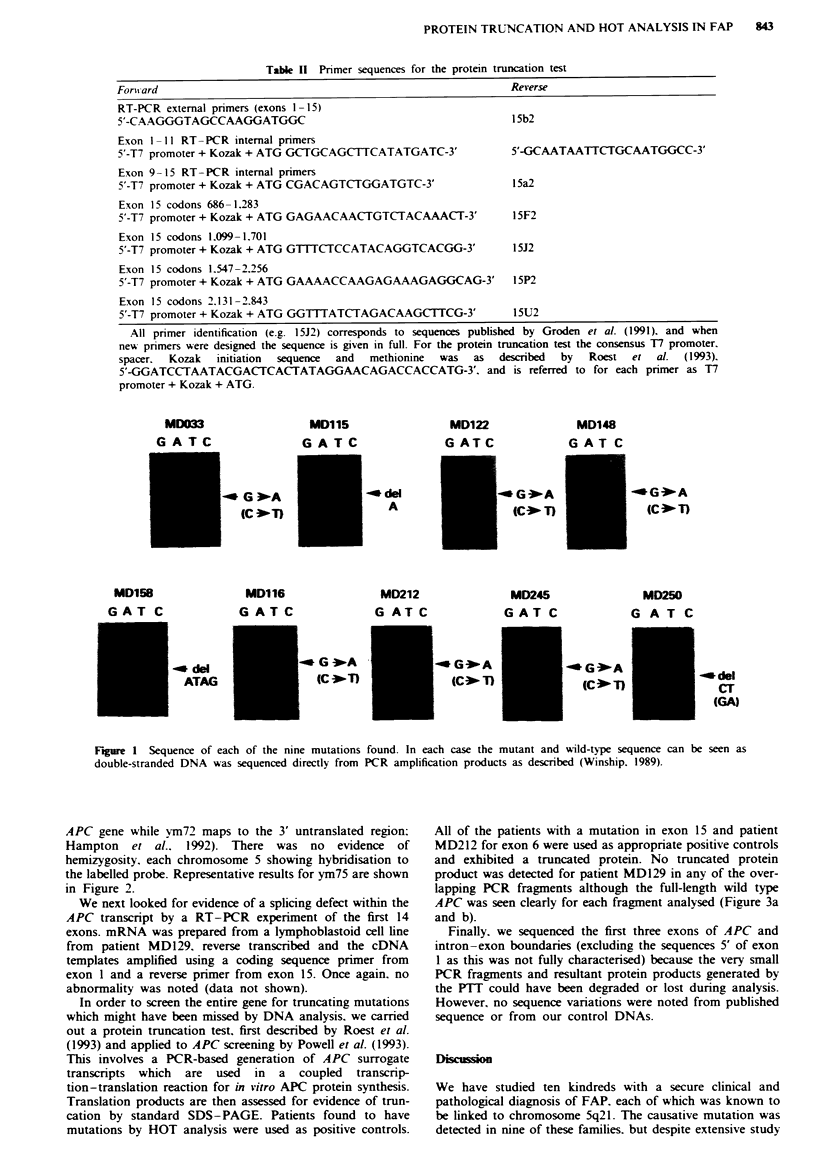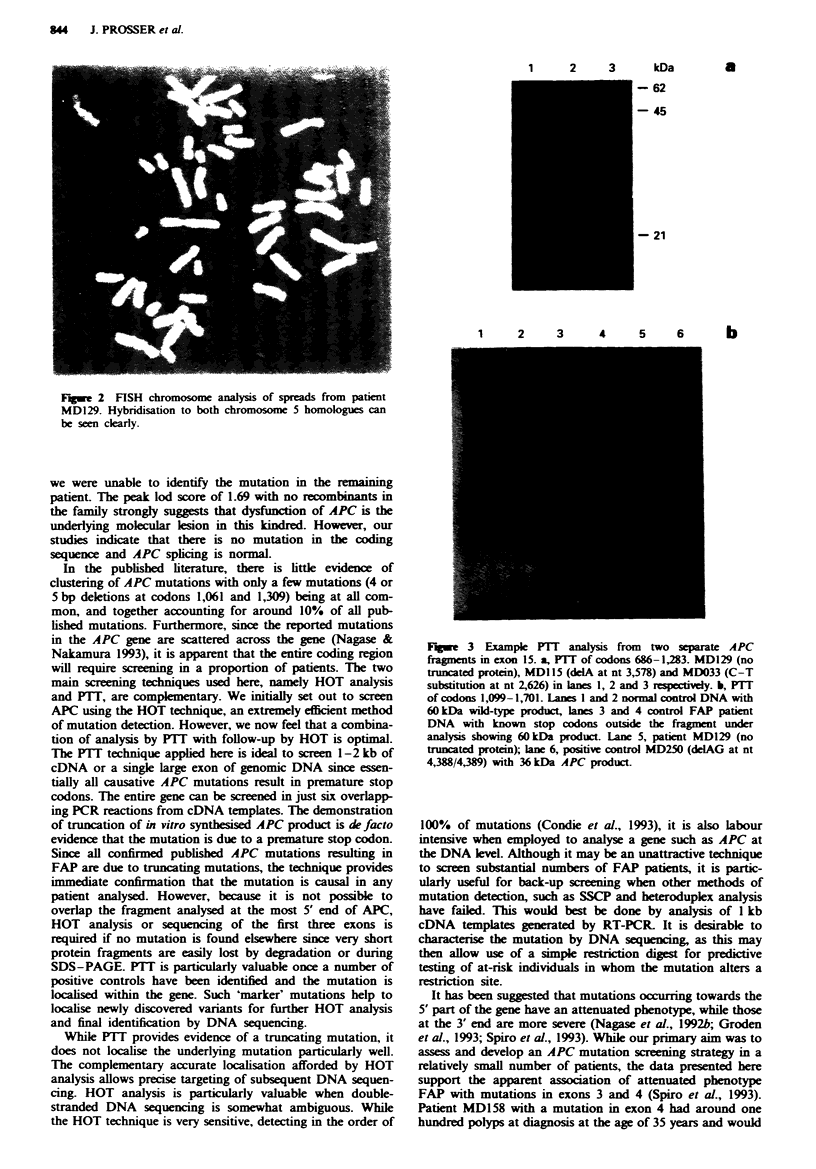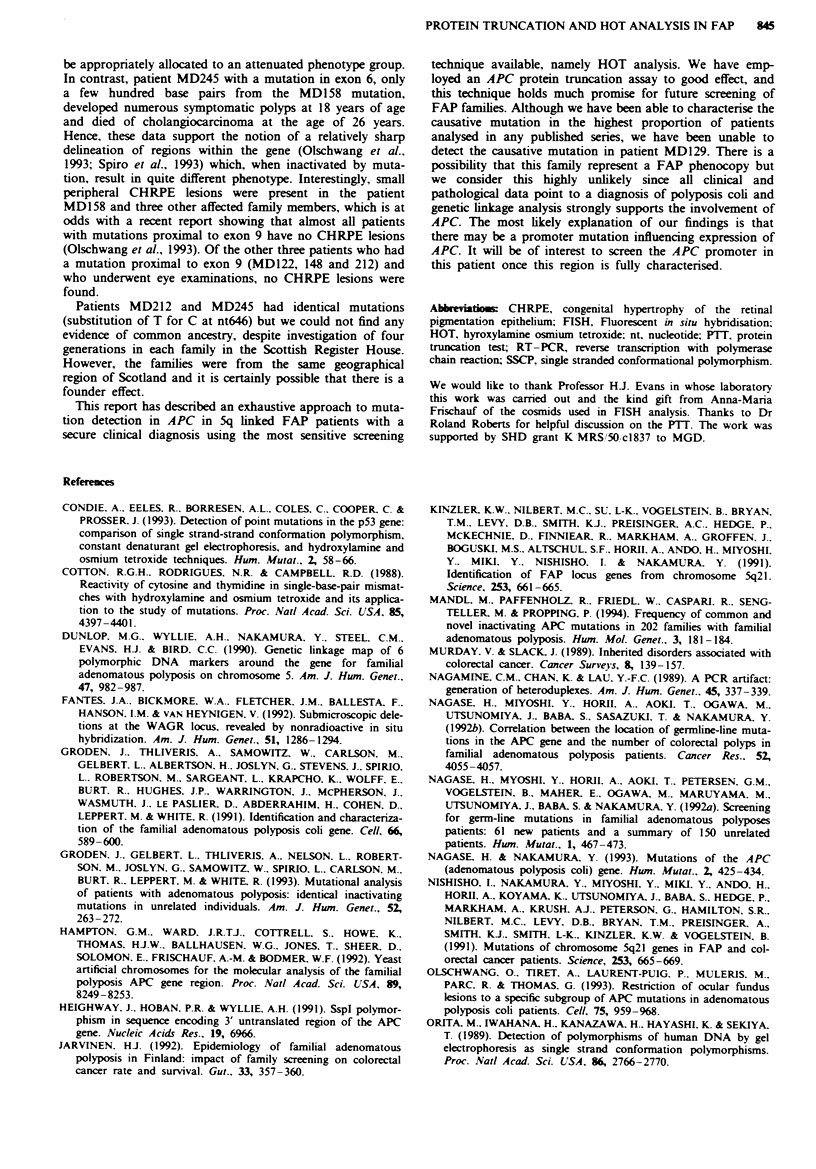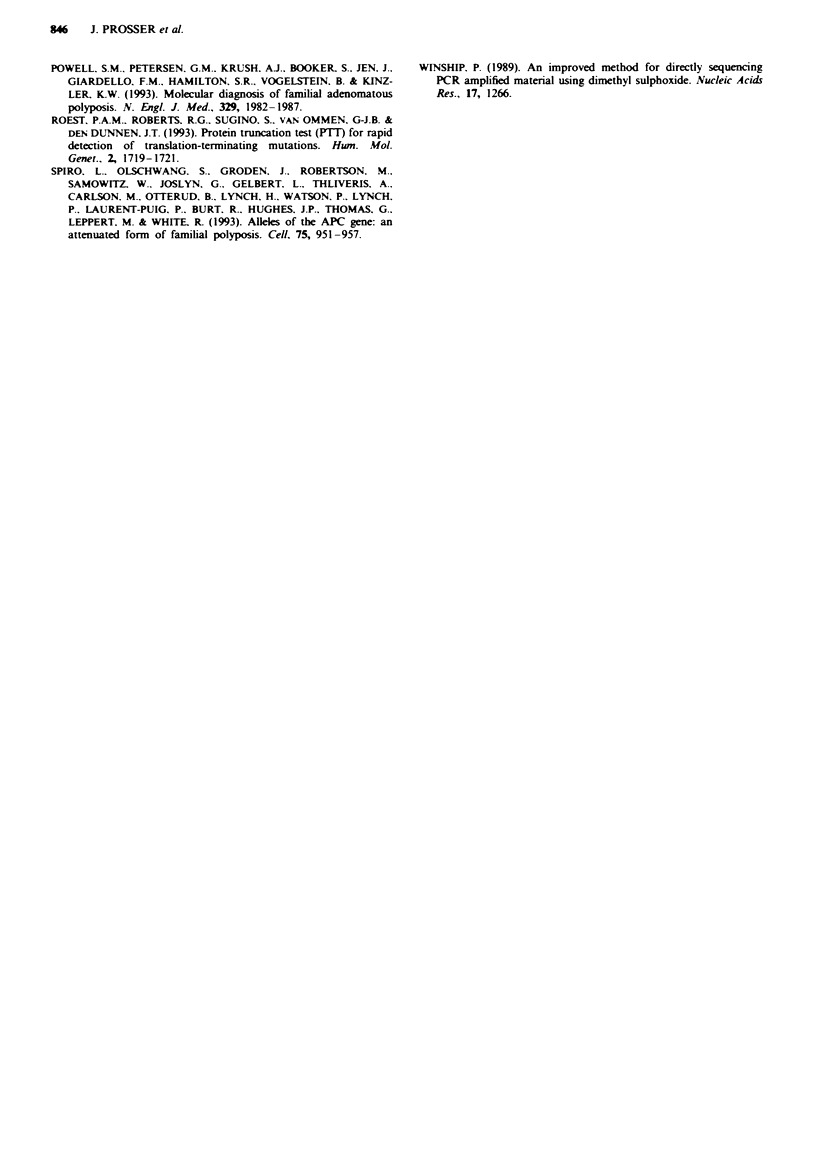# APC mutation analysis by chemical cleavage of mismatch and a protein truncation assay in familial adenomatous polyposis.

**DOI:** 10.1038/bjc.1994.408

**Published:** 1994-11

**Authors:** J. Prosser, A. Condie, M. Wright, J. M. Horn, J. A. Fantes, A. H. Wyllie, M. G. Dunlop

**Affiliations:** MRC Human Genetics Unit, Western General Hospital, Edinburgh, UK.

## Abstract

**Images:**


 © Macmillan Press Ltd., 1994

### APC mutation analysis by chemical cleavage of mismatch and a protein truncation assay in familial adenomatous polyposis

J. Prosser[1], A. Condie[1], M. Wright, J.M. Horn, J.A. Fantes, A.H. Wyllie[2] & M.G. Dunlop[3]

[1]*MRC Human Genetics Unit, Western General Hospital, Crewe Road, Edinburgh EH4 2XU, UK;* [2]*Cancer Research Campaign Laboratories, Department of Pathology, Edinburgh University, Edinburgh EH8 9AG, UK; and* [3]*Edinburgh University Department of Clinical Surgery, Royal Infirmary, Edinburgh EH3 9YW, UK.*

**Summary** Overall, the causative *APC* mutation has been identified in only 30% of the patients with familial adenomatous polyposis (FAP) who have been included in studies reported in the literature. In order to determine the true frequency of detectable APC mutations, we set out to search exhaustively the entire coding region of APC for causative mutations in ten patients with classical FAP from Scottish kindreds shown to be linked to 5q markers. Chemical cleavage of mismatch analysis was employed as the initial screening technique. Mutations were confirmed by direct DNA sequencing and shown to generate a premature stop codon by an *in vitro* protein synthesis assay. Mutations resulting in a premature stop codon either by base substitution or by frameshift were identified in nine families. Although the remaining kindred was linked to intragenic *APC* markers with a lodscore of 1.69 at $Z_{max} = 0.0$, further analysis of DNA, RNA and chromosome spreads from the proband failed to detect any abnormality. This was despite employing single-strand conformation polymorphism (SSCP) analysis, heteroduplex analysis, DNA sequencing, reverse transcription–polymerase chain reaction (RT–PCR) analysis for splicing defects, a protein truncation test encompassing the entire *APC* gene and fluorescent *in situ* hybridisation chromosome analysis (FISH). These data show that 90% of these FAP kindreds had *APC* mutations detectable by chemical cleavage of mismatch and that none of the numerous other techniques employed could detect the mutation in the remaining kindred. This study shows the value of screening the *APC* gene using a combination of chemical cleavage of mismatch analysis and an *in vitro* protein truncation test.

Familial adenomatous polyposis (FAP) is an autosomal dominant heritable disorder with a population frequency of around 1:7,000. Penetrance is almost 100% and the syndrome is characterised by the development of hundreds or thousands of colorectal polyps during the second decade of life. The presence of more than 100 adenomatous polyps is diagnostic of FAP. Progression of one or more of the polyps to carcinoma is almost inevitable unless prophylactic surgical excision of the large bowel is performed (Murday *et al.*, 1989). The clinical manifestations of FAP are a result of mutations in *APC*, a gene located on chromosome 5q21–q22 and for which the entire coding sequence is now known (Groden *et al.*, 1991; Kinzler *et al.*, 1991; Nishishio *et al.*, 1991). Nonetheless, of over 800 FAP patients reported in the world literature, the overall frequency of identifying the causative mutation is only 30% (Nagase *et al.*, 1992*a*; Nagase & Nakamura, 1993; Mandl *et al.*, 1994) and ranges from 21% (Mandl *et al.*, 1994) to 67% (Nagase *et al.*, 1992*a*). This may reflect any one or more of the following: the selection criteria for the diagnosis of FAP, the sensitivity and robustness of the mutation detection technique employed, the assiduousness of the search for mutations, the presence of causative gene alterations influencing *APC* expression outwith the coding sequence and the possibility of genetic heterogeneity in FAP.

Presymptomatic colonic screening of at-risk relatives and appropriate prophylactic surgery considerably reduces morbidity and mortality from FAP (Jarvinen, 1992). Since reliable diagnosis by identification of constitutional mutations in the *APC* gene will obviate the need for frequent colonoscopy of relatives at risk, it is of substantial clinical importance to know what proportion of patients carry constitutional *APC* alterations and with what reliance these can be detected. With this in mind, we undertook an exhaustive examination of the *APC* gene in a group of ten unrelated FAP patients who had both a secure clinical diagnosis of FAP and who were from kindreds in which there was evidence of genetic linkage to *APC* markers. Mutation detection in this study was exhaustive, employing chemical cleavage of mismatch analysis (HOT, for hydroxylamine and osmium tetroxide used in the technique) as the primary screening procedure. In the patient in whom no variant was found by HOT analysis, we performed further, extensive studies in an attempt to identify the underlying *APC* mutation, including SSCP, heteroduplex analysis, DNA sequencing, RT–PCR analysis for splicing defects, fluorescent *in situ* hybridisation (FISH) chromosome analysis and a modification of a protein truncation assay (PTT) (Powell *et al.*, 1993; Roest *et al.*, 1993) using a coupled transcription–translation system.

Correspondence: M.G. Dunlop.
Received 25 March 1994; and in revised form 24 June 1994.

### Materials and methods

Ten patients with FAP (i.e. > 100 adenomatous polyps noted in the colonic resection specimen) from families which had previously shown some evidence of genetic linkage to 5q markers were selected at random from Scottish families collected as part of a national registry. DNA samples and/or lymphoblastoid cell lines for RNA studies were obtained. DNA and RNA purification was performed to standard protocols. The clinical phenotype and particular mutation found are detailed for each subject in Table I. The measure of density of polyps was divided into dense and sparse on subjective assessment of clinical photographs or clinical inspection of the colon without a numerical definition since such a definition would be dependent on the assiduousness of the search for polyps.

### *DNA sequencing*

PCR fragments shown to contain a variant band on mutation screening by HOT analysis was reamplified with the same primers and double-stranded sequencing performed as described (Winship, 1989) using either the forward or the reverse primer to detect the causative *APC* mutation.

*HOT analysis*

The HOT technique, which detects in the region of 100% of mutations, was used on DNA fragments PCR amplified from blood lymphocyte DNA as previously described (Cotton *et al.*, 1988; Condie *et al.*, 1993). The gene was amplified exon by exon according to published primers (Groden *et al.*, 1991). Fourteen overlapping fragments covered exons 1–14 and varied in size from 173 to 458 bp. In exon 15 the size of the amplified fragments was doubled, resulting in 11 instead of 23 fragments, ranging in size from 542 to 870 bp. Twenty-five PCR amplified fragments covered the entire gene from 20 bp 5′ of the initiating ATG to 31 bp 3′ of translation termination. The 5′ primer of exon 3 was entirely within exon 3 and of exon 4 extended into exon 4. Mutations under these primers would not be detected, nor would splicing mutations involving the donor sites of introns 2 and 3, but these regions constitute a very small proportion of the entire gene.

*Single-stranded conformational polymorphism (SSCP) analysis*

PCR amplification of all *APC* fragments was carried out as described by Groden *et al.* (1991), incorporating 0.25 μCi of [$^{32}$P]CTP for each reaction. SSCP was carried out as previously described (Orita *et al.*, 1989; Groden *et al.*, 1991). PCR products were denatured and run on 6% polyacrylamide gels with 10% glycerol at room temperature under non-denaturing conditions. Gels were dried and autoradiography performed for 6–24 h.

*Heteroduplex analysis*

PCR amplification of all APC fragments was carried out as described for SSCP analysis and heteroduplex analysis performed as described (Nagamine *et al.*, 1989). Autoradiographs were assessed after 6–24 h).

*Protein truncation test (PTT)*

PCR amplification was used to introduce the 17 bp consensus T7 promoter sequence and a mammalian translation initiation sequence in-frame with unique *APC* sequence as previously described (Powell *et al.*, 1993). Unique primers for most of exon 15 were as described by Powell *et al.* (segments 3–5), but we modified primers for exons 1–14 in order to analyse cDNA templates for exons 1–14 in two overlapping reactions. We also designed the forward primer for the most 5′ part of exon 15 to be closer to the splice site. Therefore, a total of six forward primer sets were designed in the following way: T7 consensus promoter sequence–spacer–Kozak consensus sequence–ATG-unique *APC* sequence in-frame with the ATG in order to allow overlapping fragments of the entire gene when suitable reverse primers from the published sequences (Groden *et al.*, 1991) were selected.

Exons 1–14 were amplified from cDNA which had been reverse transcribed from lyphoblastoid cell line RNA using a two-stage nested PCR technique with the internal primer bearing the T7 promoter and initiation sequences. Primer sequences used in the PTT are shown in Table II. PCR products were then used without purification in a coupled transcription–translation reaction (Promega UK) incorporating 40 μCi of [$^{35}$S]methionine according to the manufacturer's instructions. The resultant products were diluted in buffer, boiled and analysed by 8%, 10% and 12% SDS–PAGE gels. Gels were washed in fix and autoradiography performed at room temperature overnight.

*Fluorescent in situ hybridisation (FISH)*

Biotinylated cosmids were hybridised to metaphase slides from short-term peripheral blood cultures from the patient and hybridised probe was detected by alternate layers of avidin–FITC, biotinylated anti-avidin and avidin–FITC as previously described (Fantes *et al.*, 1992). Twenty metaphases were examined for each probe for the presence of signal on both chromosomes 5 using a BioRad laser scanning confocal microscope.

**Results**

Using HOT analysis and sequencing of variants, we found inactivating constitutional mutations in nine patients (90%). Mutations were found in exons 4, 6 (three), 8, 13 and 15 (three) Table I and Figure 1). Three mutations constituted deletions (of 1 bp, 2 bp and 4 bp) leading to subsequent stop codons by frameshift. Six mutations were C–T transitions at CpG sites, in each case changing an arginine to a stop. One-third of the mutations were found in exon 15, although none was found in mutational hotspot regions of the gene (Δ1061 and Δ1309 in exon 15) (Nagase & Nakamura, 1993). No double mutations were detected despite analysis of the entire *APC* gene in every patient.

We were unable to detect any alteration in the *APC* gene in one FAP patient (MD129). The kindred of this patient was relatively small and DNA was not retrievable from some deceased family members. However, linkage analysis using intragenic *APC* polymorphisms achieved a peak lod score of 1.69 at $\theta = 0.0$, thereby substantially excluding the possibility of another non-5q-linked locus being involved. HOT analysis had shown no heterozygosity at any of the known *APC* polymorphic sites even though more than 20 intragenic restriction site polymorphisms (summarised by Nagase *et al.*, 1992*b*) were screened. In addition, flanking linked markers EF544 and L562 (Dunlop *et al.*, 1990) showed no heterozygosity and a 3′ PCR-amplifiable *SspI* polymorphism (Heighway *et al.*, 1991) was found to be homozygous. SSCP and heteroduplex analysis of each segment of *APC* in patient MD129 also failed to reveal any mutation or polymorphisms.

In view of the persistent evidence of homozygosity at every locus examined, we considered whether the patient might be hemizygous at the *APC* locus. Karyotype analysis of G-banded chromosome spreads from peripheral lymphocytes identified two normal chromosome 5 homologues. Therefore, fluorescent *in situ* hybridisation (FISH) was carried out using cosmids that map to the *APC* locus (ym75 maps within the

**Table I** Mutation and clinical features for each patient

| *Patient* | *Age at diagnosis* | *Polyp density* | *Extracolonic features* | *Mutation* | *Exon* |
|---|---|---|---|---|---|
| MD115 | 18 | Dense | None | Del A at nt3578 | 15 |
| MD122 | 18 | Dense | Epidermoid cysts | CGA->TGA at nt904 | 8 |
| MD129 | 23 | Dense | None | Not found | N A |
| MD148 | 27 | Dense | Epidermoid cysts | CGA->at nt694 | 6 |
| MD033 | 18 | Dense | None | CGA->at nt2626 | 15 |
| MD158 | 35 | Sparse | Epidermoid cysts | DelATAG at nt509/512 | 4 |
| MD166 | 39 | Dense | None | CGA->TGA at nt1660 | 13 |
| MD212 | 45 | Sparse | Osteomas | CGA->TGA at nt646 | 6 |
| MD245 | 18 | Dense | Osteomas<br>Cholangiocarcinoma | CGA->TGA at nt646 | 6 |
| MD250 | 25 | Dense | Osteomas, desmoids | DelAG at nt4388/4389 | 15 |

**Table II** Primer sequences for the protein truncation test

| *Forward* | *Reverse* |
|---|---|
| RT-PCR external primers (exons 1-15) 5′-CAAGGGTAGCCAAGGATGGC | 15b2 |
| Exon 1-11 RT-PCR internal primers 5′-T7 promoter + Kozak + ATG GCTGCAGCTTCATATGATC-3′ | 5′-GCAATAATTCTGCAATGGCC-3′ |
| Exon 9-15 RT-PCR internal primers 5′-T7 promoter + Kozak + ATG CGACAGTCTGGATGTC-3′ | 15a2 |
| Exon 15 codons 686-1.283 5′-T7 promoter + Kozak + ATG GAGAACAACTGTCTACAAACT-3′ | 15F2 |
| Exon 15 codons 1.099-1.701 5′-T7 promoter + Kozak + ATG GTTTCTCCATACAGGTCACGG-3′ | 15J2 |
| Exon 15 codons 1.547-2.256 5′-T7 promoter + Kozak + ATG GAAAACCAAGAGAAAGAGGCAG-3′ | 15P2 |
| Exon 15 codons 2.131-2.843 5′-T7 promoter + Kozak + ATG GGTTTATCTAGACAAGCTTCG-3′ | 15U2 |

All primer identification (e.g. 15J2) corresponds to sequences published by Groden *et al.* (1991). and when new primers were designed the sequence is given in full. For the protein truncation test the consensus T7 promoter. spacer. Kozak initiation sequence and methionine was as described by Roest *et al.* (1993). 5′-GGATCCTAATACGACTCACTATAGGAACAGACCACCATG-3′. and is referred to for each primer as T7 promoter + Kozak + ATG.

**Figure 1** Sequence of each of the nine mutations found. In each case the mutant and wild-type sequence can be seen as double-stranded DNA was sequenced directly from PCR amplification products as described (Winship, 1989).

*APC* gene while ym72 maps to the 3′ untranslated region; Hampton *et al.*, 1992). There was no evidence of hemizygosity, each chromosome 5 showing hybridisation to the labelled probe. Representative results for ym75 are shown in Figure 2.

We next looked for evidence of a splicing defect within the *APC* transcript by a RT-PCR experiment of the first 14 exons. mRNA was prepared from a lymphoblastoid cell line from patient MD129, reverse transcribed and the cDNA templates amplified using a coding sequence primer from exon 1 and a reverse primer from exon 15. Once again, no abnormality was noted (data not shown).

In order to screen the entire gene for truncating mutations which might have been missed by DNA analysis, we carried out a protein truncation test, first described by Roest *et al.* (1993) and applied to *APC* screening by Powell *et al.* (1993). This involves a PCR-based generation of *APC* surrogate transcripts which are used in a coupled transcription-translation reaction for *in vitro* APC protein synthesis. Translation products are then assessed for evidence of truncation by standard SDS-PAGE. Patients found to have mutations by HOT analysis were used as positive controls. All of the patients with a mutation in exon 15 and patient MD212 for exon 6 were used as appropriate positive controls and exhibited a truncated protein. No truncated protein product was detected for patient MD129 in any of the overlapping PCR fragments although the full-length wild type *APC* was seen clearly for each fragment analysed (Figure 3a and b).

Finally, we sequenced the first three exons of *APC* and intron-exon boundaries (excluding the sequences 5′ of exon 1 as this was not fully characterised) because the very small PCR fragments and resultant protein products generated by the PTT could have been degraded or lost during analysis. However, no sequence variations were noted from published sequence or from our control DNAs.

### Discussion

We have studied ten kindreds with a secure clinical and pathological diagnosis of FAP, each of which was known to be linked to chromosome 5q21. The causative mutation was detected in nine of these families, but despite extensive study

**Figure 2** FISH chromosome analysis of spreads from patient MD129. Hybridisation to both chromosome 5 homologues can be seen clearly.

**Figure 3** Example PTT analysis from two separate *APC* fragments in exon 15. **a**, PTT of codons 686–1,283. MD129 (no truncated protein), MD115 (delA at nt 3,578) and MD033 (C–T substitution at nt 2,626) in lanes 1, 2 and 3 respectively. **b**, PTT of codons 1,099–1,701. Lanes 1 and 2 normal control DNA with 60 kDa wild-type product, lanes 3 and 4 control FAP patient DNA with known stop codons outside the fragment under analysis showing 60 kDa product. Lane 5, patient MD129 (no truncated protein); lane 6, positive control MD250 (delAG at nt 4,388/4,389) with 36 kDa *APC* product.

we were unable to identify the mutation in the remaining patient. The peak lod score of 1.69 with no recombinants in the family strongly suggests that dysfunction of *APC* is the underlying molecular lesion in this kindred. However, our studies indicate that there is no mutation in the coding sequence and *APC* splicing is normal.

In the published literature, there is little evidence of clustering of *APC* mutations with only a few mutations (4 or 5 bp deletions at codons 1,061 and 1,309) being at all common, and together accounting for around 10% of all published mutations. Furthermore, since the reported mutations in the *APC* gene are scattered across the gene (Nagase & Nakamura 1993), it is apparent that the entire coding region will require screening in a proportion of patients. The two main screening techniques used here, namely HOT analysis and PTT, are complementary. We initially set out to screen APC using the HOT technique, an extremely efficient method of mutation detection. However, we now feel that a combination of analysis by PTT with follow-up by HOT is optimal. The PTT technique applied here is ideal to screen 1–2 kb of cDNA or a single large exon of genomic DNA since essentially all causative *APC* mutations result in premature stop codons. The entire gene can be screened in just six overlapping PCR reactions from cDNA templates. The demonstration of truncation of *in vitro* synthesised *APC* product is *de facto* evidence that the mutation is due to a premature stop codon. Since all confirmed published *APC* mutations resulting in FAP are due to truncating mutations, the technique provides immediate confirmation that the mutation is causal in any patient analysed. However, because it is not possible to overlap the fragment analysed at the most 5′ end of APC, HOT analysis or sequencing of the first three exons is required if no mutation is found elsewhere since very short protein fragments are easily lost by degradation or during SDS–PAGE. PTT is particularly valuable once a number of positive controls have been identified and the mutation is localised within the gene. Such 'marker' mutations help to localise newly discovered variants for further HOT analysis and final identification by DNA sequencing.

While PTT provides evidence of a truncating mutation, it does not localise the underlying mutation particularly well. The complementary accurate localisation afforded by HOT analysis allows precise targeting of subsequent DNA sequencing. HOT analysis is particularly valuable when double-stranded DNA sequencing is somewhat ambiguous. While the HOT technique is very sensitive, detecting in the order of 100% of mutations (Condie *et al.*, 1993), it is also labour intensive when employed to analyse a gene such as *APC* at the DNA level. Although it may be an unattractive technique to screen substantial numbers of FAP patients, it is particularly useful for back-up screening when other methods of mutation detection, such as SSCP and heteroduplex analysis have failed. This would best be done by analysis of 1 kb cDNA templates generated by RT-PCR. It is desirable to characterise the mutation by DNA sequencing, as this may then allow use of a simple restriction digest for predictive testing of at-risk individuals in whom the mutation alters a restriction site.

It has been suggested that mutations occurring towards the 5′ part of the gene have an attenuated phenotype, while those at the 3′ end are more severe (Nagase *et al.*, 1992*b*; Groden *et al.*, 1993; Spiro *et al.*, 1993). While our primary aim was to assess and develop an *APC* mutation screening strategy in a relatively small number of patients, the data presented here support the apparent association of attenuated phenotype FAP with mutations in exons 3 and 4 (Spiro *et al.*, 1993). Patient MD158 with a mutation in exon 4 had around one hundred polyps at diagnosis at the age of 35 years and would be appropriately allocated to an attenuated phenotype group. In contrast, patient MD245 with a mutation in exon 6, only a few hundred base pairs from the MD158 mutation, developed numerous symptomatic polyps at 18 years of age and died of cholangiocarcinoma at the age of 26 years. Hence, these data support the notion of a relatively sharp delineation of regions within the gene (Olschwang *et al.*, 1993; Spiro *et al.*, 1993) which, when inactivated by mutation, result in quite different phenotype. Interestingly, small peripheral CHRPE lesions were present in the patient MD158 and three other affected family members, which is at odds with a recent report showing that almost all patients with mutations proximal to exon 9 have no CHRPE lesions (Olschwang *et al.*, 1993). Of the other three patients who had a mutation proximal to exon 9 (MD122, 148 and 212) and who underwent eye examinations, no CHRPE lesions were found.

Patients MD212 and MD245 had identical mutations (substitution of T for C at nt646) but we could not find any evidence of common ancestry, despite investigation of four generations in each family in the Scottish Register House. However, the families were from the same geographical region of Scotland and it is certainly possible that there is a founder effect.

This report has described an exhaustive approach to mutation detection in *APC* in 5q linked FAP patients with a secure clinical diagnosis using the most sensitive screening technique available, namely HOT analysis. We have employed an *APC* protein truncation assay to good effect, and this technique holds much promise for future screening of FAP families. Although we have been able to characterise the causative mutation in the highest proportion of patients analysed in any published series, we have been unable to detect the causative mutation in patient MD129. There is a possibility that this family represent a FAP phenocopy but we consider this highly unlikely since all clinical and pathological data point to a diagnosis of polyposis coli and genetic linkage analysis strongly supports the involvement of *APC*. The most likely explanation of our findings is that there may be a promoter mutation influencing expression of *APC*. It will be of interest to screen the *APC* promoter in this patient once this region is fully characterised.

**Abbreviations:** CHRPE, congenital hypertrophy of the retinal pigmentation epithelium; FISH, Fluorescent *in situ* hybridisation; HOT, hyroxylamine osmium tetroxide; nt, nucleotide; PTT, protein truncation test; RT–PCR, reverse transcription with polymerase chain reaction; SSCP, single stranded conformational polymorphism.

We would like to thank Professor H.J. Evans in whose laboratory this work was carried out and the kind gift from Anna-Maria Frischauf of the cosmids used in FISH analysis. Thanks to Dr Roland Roberts for helpful discussion on the PTT. The work was supported by SHD grant K/MRS/50/c1837 to MGD.

**References**

CONDIE, A., EELES, R., BORRESEN, A.L., COLES, C., COOPER, C. & PROSSER, J. (1993). Detection of point mutations in the p53 gene: comparison of single strand-strand conformation polymorphism, constant denaturant gel electrophoresis, and hydroxylamine and osmium tetroxide techniques. *Hum. Mutat.*, **2**, 58–66.

COTTON, R.G.H., RODRIGUES, N.R. & CAMPBELL, R.D. (1988). Reactivity of cytosine and thymidine in single-base-pair mismatches with hydroxylamine and osmium tetroxide and its application to the study of mutations. *Proc. Natl Acad. Sci. USA*, **85**, 4397–4401.

DUNLOP, M.G., WYLLIE, A.H., NAKAMURA, Y., STEEL, C.M., EVANS, H.J. & BIRD, C.C. (1990). Genetic linkage map of 6 polymorphic DNA markers around the gene for familial adenomatous polyposis on chromosome 5. *Am. J. Hum. Genet.*, **47**, 982–987.

FANTES, J.A., BICKMORE, W.A., FLETCHER, J.M., BALLESTA, F., HANSON, I.M. & VAN HEYNIGEN, V. (1992). Submicroscopic deletions at the WAGR locus, revealed by nonradioactive in situ hybridization. *Am. J. Hum. Genet.*, **51**, 1286–1294.

GRODEN, J., THLIVERIS, A., SAMOWITZ, W., CARLSON, M., GELBERT, L., ALBERTSON, H., JOSLYN, G., STEVENS, J., SPIRIO, L., ROBERTSON, M., SARGEANT, L., KRAPCHO, K., WOLFF, E., BURT, R., HUGHES, J.P., WARRINGTON, J., MCPHERSON, J., WASMUTH, J., LE PASLIER, D., ABDERRAHIM, H., COHEN, D., LEPPERT, M. & WHITE, R. (1991). Identification and characterization of the familial adenomatous polyposis coli gene. *Cell*, **66**, 589–600.

GRODEN, J., GELBERT, L., THLIVERIS, A., NELSON, L., ROBERTSON, M., JOSLYN, G., SAMOWITZ, W., SPIRIO, L., CARLSON, M., BURT, R., LEPPERT, M. & WHITE, R. (1993). Mutational analysis of patients with adenomatous polyposis: identical inactivating mutations in unrelated individuals. *Am. J. Hum. Genet.*, **52**, 263–272.

HAMPTON, G.M., WARD, J.R.T.J., COTTRELL, S., HOWE, K., THOMAS, H.J.W., BALLHAUSEN, W.G., JONES, T., SHEER, D., SOLOMON, E., FRISCHAUF, A.-M. & BODMER, W.F. (1992). Yeast artificial chromosomes for the molecular analysis of the familial polyposis APC gene region. *Proc. Natl Acad. Sci. USA*, **89**, 8249–8253.

HEIGHWAY, J., HOBAN, P.R. & WYLLIE, A.H. (1991). SspI polymorphism in sequence encoding 3′ untranslated region of the APC gene. *Nucleic Acids Res.*, **19**, 6966.

JARVINEN, H.J. (1992). Epidemiology of familial adenomatous polyposis in Finland: impact of family screening on colorectal cancer rate and survival. *Gut.*, **33**, 357–360.

KINZLER, K.W., NILBERT, M.C., SU, L-K., VOGELSTEIN, B., BRYAN, T.M., LEVY, D.B., SMITH, K.J., PREISINGER, A.C., HEDGE, P., MCKECHNIE, D., FINNIEAR, R., MARKHAM, A., GROFFEN, J., BOGUSKI, M.S., ALTSCHUL, S.F., HORII, A., ANDO, H., MIYOSHI, Y., MIKI, Y., NISHISHO, I. & NAKAMURA, Y. (1991). Identification of FAP locus genes from chromosome 5q21. *Science*, **253**, 661–665.

MANDL, M., PAFFENHOLZ, R., FRIEDL, W., CASPARI, R., SENGTELLER, M. & PROPPING, P. (1994). Frequency of common and novel inactivating APC mutations in 202 families with familial adenomatous polyposis. *Hum. Mol. Genet.*, **3**, 181–184.

MURDAY, V. & SLACK, J. (1989). Inherited disorders associated with colorectal cancer. *Cancer Surveys*, **8**, 139–157.

NAGAMINE, C.M., CHAN, K. & LAU, Y.-F.C. (1989). A PCR artifact: generation of heteroduplexes. *Am. J. Hum. Genet.*, **45**, 337–339.

NAGASE, H., MIYOSHI, Y., HORII, A., AOKI, T., OGAWA, M., UTSUNOMIYA, J., BABA, S., SASAZUKI, T. & NAKAMURA, Y. (1992*b*). Correlation between the location of germline-line mutations in the APC gene and the number of colorectal polyps in familial adenomatous polyposis patients. *Cancer Res.*, **52**, 4055–4057.

NAGASE, H., MYOSHI, Y., HORII, A., AOKI, T., PETERSEN, G.M., VOGELSTEIN, B., MAHER, E., OGAWA, M., MARUYAMA, M., UTSUNOMIYA, J., BABA, S. & NAKAMURA, Y. (1992*a*). Screening for germ-line mutations in familial adenomatous polyposes patients: 61 new patients and a summary of 150 unrelated patients. *Hum. Mutat.*, **1**, 467–473.

NAGASE, H. & NAKAMURA, Y. (1993). Mutations of the *APC* (adenomatous polyposis coli) gene. *Hum. Mutat.*, **2**, 425–434.

NISHISHO, I., NAKAMURA, Y., MIYOSHI, Y., MIKI, Y., ANDO, H., HORII, A., KOYAMA, K., UTSUNOMIYA, J., BABA, S., HEDGE, P., MARKHAM, A., KRUSH, A.J., PETERSON, G., HAMILTON, S.R., NILBERT, M.C., LEVY, D.B., BRYAN, T.M., PREISINGER, A., SMITH, K.J., SMITH, L-K., KINZLER, K.W. & VOGELSTEIN, B. (1991). Mutations of chromosome 5q21 genes in FAP and colorectal cancer patients. *Science*, **253**, 665–669.

OLSCHWANG, O., TIRET, A., LAURENT-PUIG, P., MULERIS, M., PARC, R. & THOMAS, G. (1993). Restriction of ocular fundus lesions to a specific subgroup of APC mutations in adenomatous polyposis coli patients. *Cell*, **75**, 959–968.

ORITA, M., IWAHANA, H., KANAZAWA, H., HAYASHI, K. & SEKIYA, T. (1989). Detection of polymorphisms of human DNA by gel electrophoresis as single strand conformation polymorphisms. *Proc. Natl Acad. Sci. USA*, **86**, 2766–2770.

POWELL, S.M., PETERSEN, G.M., KRUSH, A.J., BOOKER, S., JEN, J., GIARDELLO, F.M., HAMILTON, S.R., VOGELSTEIN, B. & KINZLER, K.W. (1993). Molecular diagnosis of familial adenomatous polyposis. *N. Engl. J. Med.*, **329**, 1982–1987.

ROEST, P.A.M., ROBERTS, R.G., SUGINO, S., VAN OMMEN, G-J.B. & DEN DUNNEN, J.T. (1993). Protein truncation test (PTT) for rapid detection of translation-terminating mutations. *Hum. Mol. Genet.*, **2**, 1719–1721.

SPIRO, L., OLSCHWANG, S., GRODEN, J., ROBERTSON, M., SAMOWITZ, W., JOSLYN, G., GELBERT, L., THLIVERIS, A., CARLSON, M., OTTERUD, B., LYNCH, H., WATSON, P., LYNCH, P., LAURENT-PUIG, P., BURT, R., HUGHES, J.P., THOMAS, G., LEPPERT, M. & WHITE, R. (1993). Alleles of the APC gene: an attenuated form of familial polyposis. *Cell*, **75**, 951–957.

WINSHIP, P. (1989). An improved method for directly sequencing PCR amplified material using dimethyl sulphoxide. *Nucleic Acids Res.*, **17**, 1266.